# Effect of Liquid Immersion Freezing Temperature on the Frozen Storage Stability of Snakehead (*Channa argus*) Fillets: Water Retention, Protein Secondary Structure, and Ice Crystal Morphology

**DOI:** 10.3390/foods15213904

**Published:** 2026-10-30

**Authors:** Xinxin Li, Shiyao Kuang, Yuyang Zhang, Tao Song, Lihui Hu, Ying Liu, Hosahalli S. Ramaswamy, Yong Yu

**Affiliations:** 1 College of Biosystems Engineering and Food Science, Zhejiang University, Hangzhou 310058, China; 2 Key Laboratory of Equipment and Informatization in Environment Controlled Agriculture, Ministry of Agriculture, Hangzhou 310058, China; 3 Department of Food Science and Agricultural Chemistry, McGill University, 21111 Lakeshore Road, St-Anne-de-Bellevue, QC H9X 3V9, Canada

**Keywords:** snakehead, liquid immersion freezing, food quality, storage, ice crystals, protein secondary structure

## Abstract

This study investigated the effects of freezing method and temperature on the quality stability of snakehead fillets during frozen storage. Fillets were subjected to air freezing (AF) and liquid immersion freezing (LIF) at −20 °C, −40 °C, and −60 °C, followed by storage at −30 °C for 6 months. Results showed that freezing caused a reduction in hardness, an increase in water loss, and significant changes in protein secondary structure compared with fresh samples. Compared with AF at the same freezing temperature, LIF produced smaller ice crystal areas and diameters, suggesting reduced freezing-induced structural damage. During storage, overall quality progressively declined, as evidenced by increased water loss, decreased hardness, pH changes, alterations in protein secondary structure, and ice crystal enlargement. Among all treatments, AF-20 exhibited the most severe quality deterioration after 6 months of storage, whereas LIF-40 and LIF-60 showed better quality retention. Although LIF-60 generated the smallest ice crystals, LIF-40 also effectively reduced thawing loss and total weight loss during storage. These findings suggest that liquid immersion freezing at appropriately low temperatures can improve the frozen storage stability of snakehead fillets, with LIF-40 and LIF-60 showing favorable quality retention during prolonged storage.

## 1. Introduction

Aquatic foods play an increasingly important role in global food supply and human nutrition. According to The State of World Fisheries and Aquaculture 2026, global production of aquatic animals reached a record 195 million tonnes in 2024 [1]. China is one of the world’s leading producers of aquatic products, with a total output of 76.57 million tonnes in 2025, including 63.24 million tonnes from aquaculture [2]. Among freshwater fish species, snakehead (*Channa argus*) is widely cultured and consumed in China because of its nutritional value, desirable flesh characteristics, and suitability for further processing. Its production in China reached approximately 595,498 tonnes in 2024, indicating considerable commercial importance [3]. However, fish muscle is highly perishable because of its high moisture content and nutrient-rich composition, making effective preservation essential for maintaining quality and extending storage life.

Freezing is one of the most widely used methods for the long-term preservation of aquatic products because low temperatures markedly slow microbial growth, enzymatic reactions, and other deteriorative processes. Nevertheless, freezing does not completely prevent quality deterioration. Ice crystal formation during freezing and subsequent recrystallization during frozen storage can alter water distribution, damage muscle structure, promote protein denaturation, and ultimately lead to increased water loss and texture deterioration [4]. Previous studies on snakehead and other freshwater fish have shown that freezing conditions strongly influence ice crystal morphology, water-holding capacity, protein properties, and textural quality [5,6].

The extent of freezing-induced damage depends largely on the freezing method and temperature. Conventional air freezing (AF) is widely used because of its operational simplicity, but its relatively low heat-transfer efficiency generally results in slower freezing and may promote the formation of larger ice crystals [5,6]. Liquid nitrogen freezing can achieve rapid freezing and limit ice crystal growth, but its operating cost and continuous cryogen requirement may restrict industrial application [7,8]. More broadly, the freezing method and freezing rate have been shown to affect water retention, protein stability, texture, and ice crystal development in frozen fish [9,10,11]. In contrast, liquid immersion freezing (LIF) enhances heat transfer through direct contact between packaged products and a low-temperature liquid refrigerant. Previous studies have demonstrated that LIF can shorten freezing time, reduce ice crystal size, improve water retention, and alleviate structural deterioration compared with conventional air freezing [5,6,12]. Recent studies have further emphasized that controlling ice crystal growth is important for maintaining protein stability, water-holding capacity, and texture during frozen storage [13,14].

Although the advantages of LIF have been increasingly recognized, most previous studies have focused on freezing characteristics, comparisons among freezing methods, or quality changes immediately after freezing and during relatively short storage periods [5,6,12]. However, the effects of different LIF temperatures on the quality stability of snakehead fillets during prolonged frozen storage have not been sufficiently investigated. In particular, a lower initial freezing temperature may generate smaller ice crystals, but this does not necessarily guarantee better quality retention during subsequent storage because ice crystal growth and recrystallization may continue after the samples are transferred to a common storage temperature. Therefore, this study compared air freezing and liquid immersion freezing at −20, −40, and −60 °C, followed by frozen storage at −30 °C for 6 months. Changes in thawing loss, cooking loss, total weight loss, hardness, pH, protein secondary structure, and ice crystal morphology were systematically evaluated, and principal component analysis was used to characterize overall differences among treatments. This study aimed to evaluate the effects of freezing method and temperature on the physicochemical and structural stability of snakehead fillets during prolonged frozen storage, providing a basis for selecting appropriate liquid immersion freezing conditions.

## 2. Materials and Methods

### 2.1. Chemicals

Reagent-grade chemicals were used throughout this investigation. Carnoy’s fluid was sourced from Yuanye Biotechnology Co., Ltd. (Hangzhou, China), while anhydrous ethanol and hematoxylin–eosin stain were obtained from Hongda Chemical & Instrument Co., Ltd. (Yiwu, China) and HaoKe Biotechnology Co., Ltd. (Hangzhou, China), respectively, both based in Hangzhou, China. Xylene and ammonia were supplied by Shanghai, China-based vendors Enamar Biotechnology Co., Ltd. (Shanghai, China). and Macklin Biochemical Co., Ltd. (Shanghai, China). Finally, hydrochloric acid and neutral balsam were provided by Sinopharm Chemical Reagent Co., Ltd. (Shanghai, China).

### 2.2. Sample Preparation

Snakehead fish specimens (mean weight: 1.5 ± 0.2 kg) were sourced fresh from the San Ding Agricultural Market in Hangzhou, China, for this study. The fish were slaughtered by the vendor at the market and transported to the laboratory within 30 min after slaughter. Upon arrival, the fish were rinsed with cold water, and the heads, skin, and bones were removed for subsequent sample preparation. Muscle sample preparation followed the method described by Diao et al. [6], with minor adjustments. Dorsal tissue sections (approximately 30 × 30 × 15 mm; ~13 g) were carefully excised and sealed in polyethylene bags after air evacuation. The samples were then pre-cooled at 4 °C in a BCD-256KF refrigerator (Haier, Qingdao, China). After pre-cooling, the samples were randomly assigned to seven experimental groups: six were subjected to different freezing protocols, and one served as the untreated control.

### 2.3. Freezing and Storage

To compare the effects of air freezing (AF) and liquid immersion freezing (LIF) at different freezing temperatures, packaged snakehead fillets were frozen using modified protocols from Yang et al. [15]. Seven experimental groups were established, including six freezing treatments at −20 °C, −40 °C, and −60 °C using AF or LIF, and one untreated fresh control group. A household refrigerator (Haier BCD-256KF, China) was maintained at −20 °C, while subzero temperatures (−40 °C/−60 °C) were achieved with an ultra-low freezer (Jiesheng DW-60 W28, Huzhou, China; operational range: −25 °C to −65 °C). Each freezer was pre-set to the target temperatures for 12 h before introducing the samples to stabilize the environment.

In the AF method, the fillet samples, sealed in bags, were directly placed into the freezer. For the LIF treatments, an aqueous ethanol solution containing 80% ethanol (*v*/*v*) was precooled to the designated freezing temperature (−20, −40, or −60 °C), after which the packaged fillets were immersed in the cooling medium. The mass ratio of the cooling medium to the packaged samples was 10:1. Core temperature monitoring was conducted using K-type thermocouples (OMEGA Engineering, Swedesboro, NJ, USA; 0.6 mm diameter), which were positioned at the geometric centers of the fillets. The freezing process was terminated when the geometric center temperature of the fillets reached the corresponding target temperature (−20, −40, or −60 °C). Following freezing, the samples were transferred to storage at −30 °C and evaluated after 0, 0.5, 1, 2, 4, and 6 months. Thus, samples frozen at −40 and −60 °C experienced a temperature increase upon transfer to storage, whereas those frozen at −20 °C experienced a further decrease in temperature. Prior to quality assessment, all specimens were allowed to equilibrate at 4 °C for 24 h, ensuring consistency following their respective storage durations.

### 2.4. Thawing, Cooking and Total Weight Loss

Thawing loss (TL), cooking loss (CL), and total weight loss (TWL) were determined using modified methodologies established by Cartagena et al. [16] and Yang et al. [8]. The frozen fillets were removed from their polyethylene bags and weighed to obtain the initial sample weight (W_0_). After thawing at 4 °C for 24 h, surface moisture was gently removed using filter paper, and the samples were reweighed (W_1_). Thawing loss was subsequently calculated using the following formula:

(1) $$\mathrm{TL}\left(\%\right)=\frac{W_{0}-W_{1}}{W_{0}}\times 100,$$

The rate of change in thawing loss during storage was determined by comparing the thawing loss of samples stored for 0 months with that of samples stored for longer periods, as follows:

(2) $$\text{The rate of change in TL}\left(\%\right)=\frac{\text{TL of storage}-\text{TL at}\;0\;\text{months}}{\text{TL at}\;0\;\text{months}}\times 100,$$

Following thawing, the samples were vacuum-sealed and subjected to thermal processing at 85 °C for 30 min. After cooking, the specimens were equilibrated at ambient temperature for 30 min. Upon equilibration, the samples were removed from the packaging, and any residual surface moisture was absorbed using filter paper. The samples were then immediately reweighed (W_2_). Cooking loss (CL) was calculated using the following formula:

(3) $$\mathrm{CL}\left(\%\right)=\frac{W_{1}-W_{2}}{W_{1}}\times 100,$$

The rate of change in cooking loss during storage was calculated based on the cooking loss of samples at 0 months of storage as follows:

(4) $$\text{The rate of change in CL}\left(\%\right)=\frac{\text{CL of storage}-\text{CL at}\;0\;\text{months}}{\text{CL at}\;0\;\text{months}}\times 100,$$

Total weight loss (TWL) was determined from the initial sample weight before thawing (W_0_) and the final sample weight after cooking (W_2_), representing the cumulative mass loss during thawing and cooking, as follows:

(5) $$\mathrm{TWL}\left(\%\right)=\frac{W_{0}-W_{2}}{W_{0}}\times 100,$$

### 2.5. Texture

Texture profile analysis (TPA) was performed using a TA-XT Plus texture analyzer (Stable Micro Systems Ltd., Godalming, UK), following the methodology outlined by Fan et al. [17]. Cuboidal specimens measuring 20 × 20 × 10 mm were subjected to a two-cycle compression test with a 5-mm cylindrical probe (P/5, flat bottom). Each cycle compressed the samples to 75% of their original height at a crosshead speed of 1.0 mm/s, with a 5-s interval between cycles. The textural properties were then derived from the recorded force–time curves.

### 2.6. pH

Fillet pH determination was performed following a modified protocol from Jiang et al. [18]. Thawed snakehead fillets were triturated, and 5 g aliquots were homogenized (FJ200-SH homogenizer, Shanghai Specimen and Model Factory, Shanghai, China) with 50 mL of deionized water for 2 min. The resulting suspensions were then centrifuged (5810R Centrifuge, Eppendorf, Hamburg, Germany; 5000 rpm for 10 min). The pH of the supernatant was measured in triplicate using a calibrated FE-20 pH meter (Mettler Toledo, Hong Kong, China), with final values representing the mean of the treatment group.

### 2.7. Raman Spectroscopy Analysis

Raman spectra of the fillet samples were acquired using a method adapted from Herrero et al. [19], with minor modifications. The thawed fillet samples were cut into 1-mm-thick slices and positioned on the stage of a LabRAM Soleil Raman spectrometer (Horiba, Palaiseau, France). A laser was focused onto the sample using a lens with a specific focal length, enabling spectral data collection. Spectra were then acquired twice within the 400–2000 cm^−1^ range, with an acquisition time of 5 s per scan. The objective lens was set to 50x magnification, and the laser wavelength was 632.8 nm. Three samples from each treatment group were analyzed to obtain the Raman spectra. The acquired spectra underwent pre-processing steps, including smoothing, baseline correction, and normalization. The processed spectra were averaged, and protein secondary structure proportions were estimated from the amide I region using PeakFit 4.12 software. The amide I region (1600–1700 cm^−1^) was used to distinguish α-helix, β-sheet, β-turn, and random coil contributions according to their characteristic wavenumber ranges [19].

### 2.8. Microstructure

The ice crystal structure of the samples was examined using an isothermal freezing substitution method, with minor modifications based on previously described protocols [5,8]. To preserve structural integrity, frozen fillet specimens were cut into approximately 4 × 4 × 4 mm cubes at −20 °C. The frozen tissue cubes were immersed in Carnoy’s fluid (60% ethanol, 30% chloroform, and 10% acetic acid, *v*/*v*) and precooled to the corresponding freezing temperature for 24 h to facilitate isothermal freeze substitution. Dehydration was carried out through sequential 30-min immersions in graded ethanol solutions (50%, 75%, 85%, 95%, and 100%). After xylene clearing (Enamar Biotechnology, Shanghai, China) and paraffin embedding (Sinopharm Reagent, Shanghai, China), 4-μm transverse sections were prepared perpendicular to the muscle fibers using a microtome. The sections were mounted on glass slides, dried at 55 °C, dewaxed with xylene, and the samples were re-dehydrated by passing through 50%, 75%, 85%, 95%, and 100% ethanol for 3 min at each concentration (Hangzhou Hongda Chemical Instrument Co., Ltd., Hangzhou, China). After dehydration, tissue sections were stained with hematoxylin for 5 min (Haoke Biotechnology, Hangzhou, China), followed by rinsing with distilled water. To ensure uniform chromogenesis, sections were differentiated in 1% HCl ethanolic solution (Sinopharm Chemical Agents, Shanghai, China) and then immersed in water for 15 min. Eosin counterstaining was applied for 1 min, followed by a final 5-min wash with distilled water. The sections were then dried and mounted under neutral resin for microscopic analysis.

Microscopic analysis was conducted using a Nikon Eclipse E100 microscope (Nikon Corporation, Tokyo, Japan), and digital image quantification was performed using Image-Pro Plus 6.0 software (Media Cybernetics, Rockville, MD, USA). Ice crystal morphology was characterized by four morphometric parameters: cross-sectional area (A), which represents the two-dimensional projection surface area; equivalent diameter (D), defined as the mean diametral measurement across crystal orientations; elongation, describing the ratio of the major to minor axis lengths; and roundness (R), which quantifies the circularity approximation, as described by Equation (6):

(6) $$R=\frac{4\pi A}{P^{2}},$$

Elongation (E) quantifies the axial ratio of ice crystal cross-sections, defined as the major-to-minor axis length proportion. Its mathematical representation is given in Equation (7):

(7) $$E=\frac{\mathrm{Ax}(\max)}{\mathrm{Ax}(\min)},$$

In this context, A represents the cross-sectional area, and P denotes the perimeter of the ice crystal’s cross-section. For image-based morphometric analysis, more than 100 ice crystal cavities were measured per slide using Image-Pro Plus software. The equivalent-diameter distributions were subsequently plotted using Origin 2018 software (OriginLab, Northampton, MA, USA).

### 2.9. Statistical Analysis

Measurements were performed in triplicate, and the results are presented as mean ± standard deviation (SD). Statistical analyses were conducted using IBM SPSS Statistics v23 (SPSS Inc., Chicago, IL, USA) and analysis of variance (ANOVA) followed by Duncan’s multiple range test to compare treatment groups at each storage time (*p* < 0.05) and complementary principal component analysis (PCA). Graphical representations were generated using Origin 2018 (OriginLab, Northampton, MA, USA).

## 3. Results and Discussion

### 3.1. Thawing, Cooking and Total Weight Loss

Moisture content plays a critical role in determining the quality of aquatic products [20]. Excessive water loss during frozen storage can adversely affect their appearance, weight, and nutritional value, ultimately diminishing consumer acceptability [21]. During the thawing process, moisture loss (drip loss) is accompanied by loss of weight as well as valuable nutrients in the exudate that results in reduced quality and market price of aquatic products [22].

As shown in Table 1, thawing loss varied among freezing treatments and storage periods. Thawing loss generally increased during frozen storage, although treatment-dependent fluctuations were observed, with values ranging from 0.26% to 2.43%. After freezing (zero storage time), snakehead fillets treated with both AF and LIF exhibited relatively low thawing losses of 0.54%, 0.38%, 0.29%, and 0.26%, with no significant differences (*p* > 0.05) among the AF-60, LIF-20, LIF-60, and LIF-40 groups. In contrast, the AF-20 group exhibited a significantly higher (*p* < 0.05) thawing loss of 1.05% even with zero storage time compared to other treatments. In most treatment groups, thawing loss reached its highest value after 4 months of storage and subsequently decreased at 6 months, whereas the AF-40 group exhibited a different trend. Similar findings were also reported in previous research on largemouth bass [12]. The reduction in thawing loss during the later storage period may be associated with changes in water distribution and retention within the muscle tissue. After 6 months of storage, thawing loss differed significantly among some freezing treatments (*p* < 0.05). After 6 months of storage, LIF-40 exhibited the numerically lowest thawing loss (0.52%), followed by LIF-60 (0.67%), with no significant difference between these two treatments (*p* > 0.05).

However, thawing loss did not decrease consistently with decreasing freezing temperature. This result suggests that further decreasing the freezing temperature does not necessarily lead to a proportional improvement in water retention during subsequent frozen storage. Although smaller ice crystals generally contribute to reduced muscle damage, thawing loss is also associated with changes in muscle structure and protein water-holding capacity [10,22]. In addition, the different thermal histories experienced by samples during transfer to the common storage temperature of −30 °C may influence ice crystal stability and water redistribution. Therefore, the relationship between freezing temperature and thawing loss may involve the combined effects of ice crystal morphology, protein structural changes, and subsequent frozen storage conditions.

Cooking loss was primarily attributed to muscle structure damage and thermal denaturation of proteins [23]. The primary proteins in fish muscle are myofibrillar proteins, which consist of myosin and actin [24]. The denaturation temperatures for myosin and actin are around 50 °C and 75 °C, respectively. A total duration of 30 min of cooking at 85 °C would be sufficient to denature the myofibrillar proteins of fish samples which, in turn, lead to reduced water-holding capacity and subsequent cooking losses [25]. As shown in Table 1, cooking loss generally increased during frozen storage, although some fluctuations were observed among treatments and storage periods. After freezing, the cooking loss in the fresh group was significantly different (*p* < 0.05) from that in the AF-20, LIF-20, and AF-40 groups, with the AF-20 group showing the highest cooking loss (16.79%). However, no significant differences (*p* > 0.05) were observed in cooking losses between the LIF-40, AF-60, and LIF-60 groups and the fresh fillets. After 6 months of storage, a lower increase in the cooking losses was observed in LIF-40 and LIF-60 groups, which may be again attributed to the high heat-transfer coefficient of liquid coolant resulting in more rapid freezing, achieving smaller ice crystals and reduced texture damage [15]. The changes in total weight loss generally followed a pattern similar to that of cooking loss. Overall, total weight loss tended to increase with prolonged frozen storage, although some fluctuations were observed among treatments and storage periods. After 6 months of storage, the LIF-40 and LIF-60 groups exhibited lower total weight loss (17.26% and 16.99%, respectively) than the other treatments, with significant differences compared with AF-20, LIF-20, and AF-40 (*p* < 0.05). These results suggest that low-temperature LIF may help improve water retention during frozen storage. It should be noted that although LIF-60 produced the smallest ice crystals, LIF-40 showed the lowest thawing loss after 6 months of storage. This suggests that extremely low freezing temperatures do not necessarily lead to better water retention during subsequent storage at −30 °C, as water retention may be influenced by multiple factors, including ice crystal morphology, muscle structure, and protein properties.

**Figure 1:**
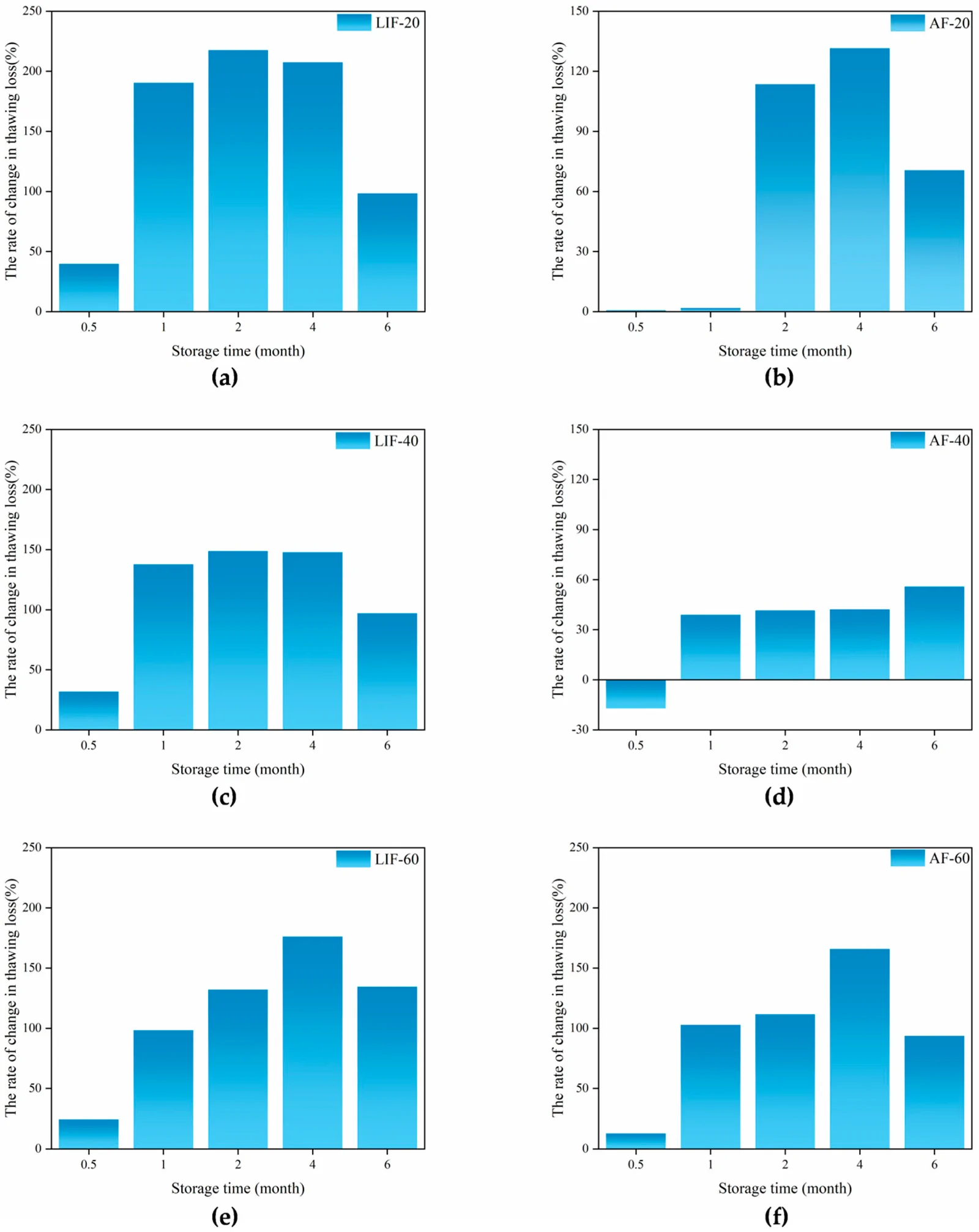
Rate of change in thawing loss of snakehead fillets during frozen storage after different freezing treatments: (**a**) LIF-20; (**b**) AF-20; (**c**) LIF-40; (**d**) AF-40; (**e**) LIF-60; and (**f**) AF-60. AF, air freezing; LIF, liquid immersion freezing; 20, 40, and 60 indicate freezing temperatures of −20, −40, and −60 °C, respectively. The rate of change in thawing loss at each storage time was calculated relative to the thawing loss at 0 months.

As shown in Figure 1, the thawing loss rate of snakehead fillets increased with storage time. Different freezing treatment groups exhibited relatively low thawing loss rates at 0.5 month of storage. By contrast, a noticeable increase in thawing loss was observed after 1 month of storage. Among the treatments, the AF-40 group exhibited a relatively small increase in thawing loss after 6 months of storage compared with its initial value. In contrast, the thawing loss rate in the AF-20 group increased significantly after 2 months, likely due to the formation of larger ice crystals during freezing at −20 °C. For the LIF-60 and AF-60 groups, the thawing loss rate rose sharply after just one month. This rapid increase may be associated with changes in ice crystal stability during frozen storage. First, the transfer from the −60 °C freezing environment to −30 °C storage may influence subsequent ice recrystallization. Second, smaller ice crystals may be more susceptible to recrystallization during storage [26]. The increase in the thawing loss rate observed in the LIF-20 and LIF-40 groups may also be associated with subsequent changes in ice crystal morphology during storage. Ice crystals formed during freezing may undergo further changes in size and morphology during subsequent frozen storage. Similar results were reported in a previous study on northern snakehead [18], where fish fillets frozen with liquid nitrogen and cooled to −60 °C exhibited a higher thawing loss rate when transferred to −20 °C storage conditions, compared to samples cooled to −18 °C.

**Figure 2:**
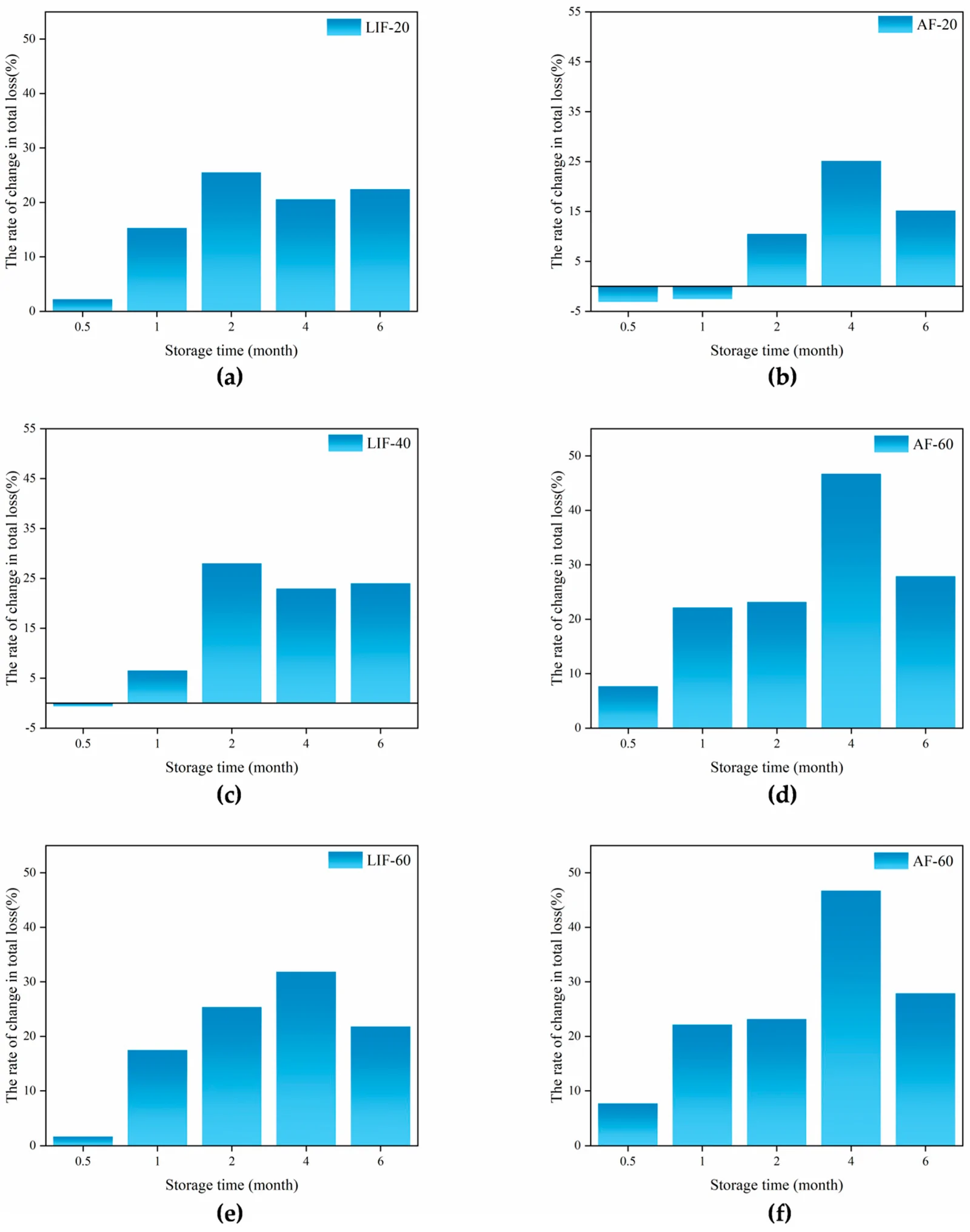
Rate of change in cooking loss of snakehead fillets during frozen storage after different freezing treatments: (**a**) LIF-20; (**b**) AF-20; (**c**) LIF-40; (**d**) AF-40; (**e**) LIF-60; and (**f**) AF-60. AF, air freezing; LIF, liquid immersion freezing; 20, 40, and 60 indicate freezing temperatures of −20, −40, and −60 °C, respectively. The rate of change in cooking loss at each storage time was calculated relative to the cooking loss at 0 months.

As shown in Figure 2, the cooking loss rate in snakehead fillets increased gradually with storage time. The samples from AF-20, LIF-20, AF-40, and LIF-40 groups exhibited lower rates of change in cooking loss during the first month of storage. In contrast, samples from these groups exhibited more pronounced changes in the rate of change in cooking loss between 2 and 6 months of storage. However, the rate of change in cooking loss of samples from the AF-60 and LIF-60 groups significantly increased after 1 month of storage. This may be associated with differences in thermal history during the transition from freezing to frozen storage, which could influence ice crystal stability and muscle structural integrity, thereby contributing to increased cooking loss [10,18].

### 3.2. Texture

**Figure 3:**
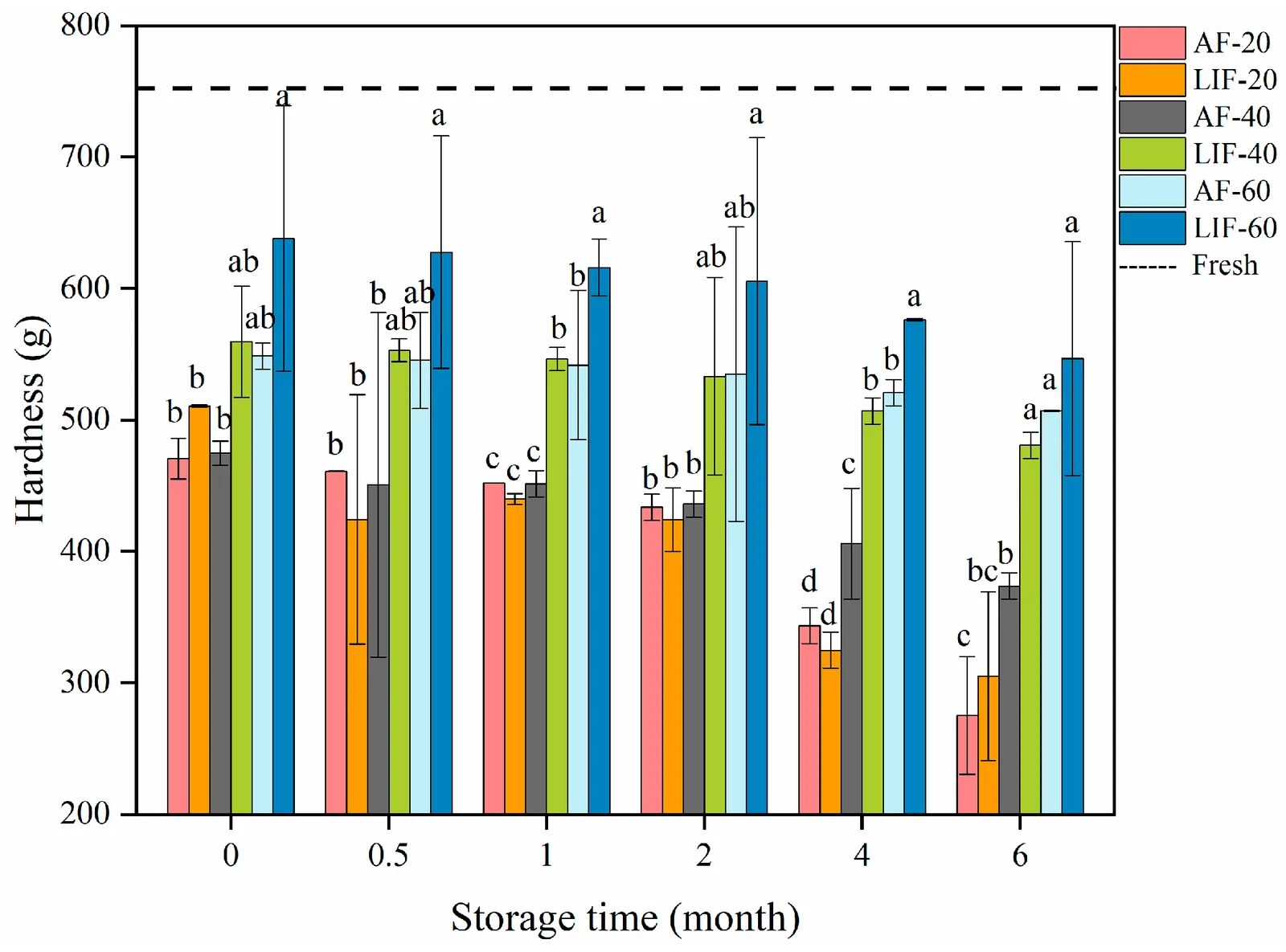
The hardness of snakehead fillets frozen by air freezing (AF) and liquid immersion freezing (LIF) at different temperatures (−20 °C, −40 °C and −60 °C) during frozen storage. The results were expressed as the mean ± standard deviation (*n* = 3), and the letters (a–d) in one storage period indicated significant differences (*p* < 0.05) between the hardness of samples treated by different freezing methods.

Hardness is an important indicator that reflects the quality of aquatic products [27]. As shown in Figure 3, the LIF-60 group exhibited the highest hardness value immediately after freezing (0 months), while the AF-20 group had the lowest. The hardness of the frozen-treated snakehead fillets significantly decreased (*p* < 0.05) compared to the fresh samples. Moreover, the hardness of samples from all groups declined over time with extended storage. After 6 months, the hardness values of the LIF-60, AF-60, and LIF-40 groups were significantly different (*p* < 0.05) from those of the AF-40, LIF-20, and AF-20 groups. This observation is consistent with previous findings for bighead carp [28]. During storage, the growth of ice crystals disrupts the integrity of muscle fibers, leading to a reduction in the hardness of the samples [18]. Additionally, protein denaturation during storage also contributes towards a decrease in the hardness value [29].

### 3.3. pH

**Figure 4:**
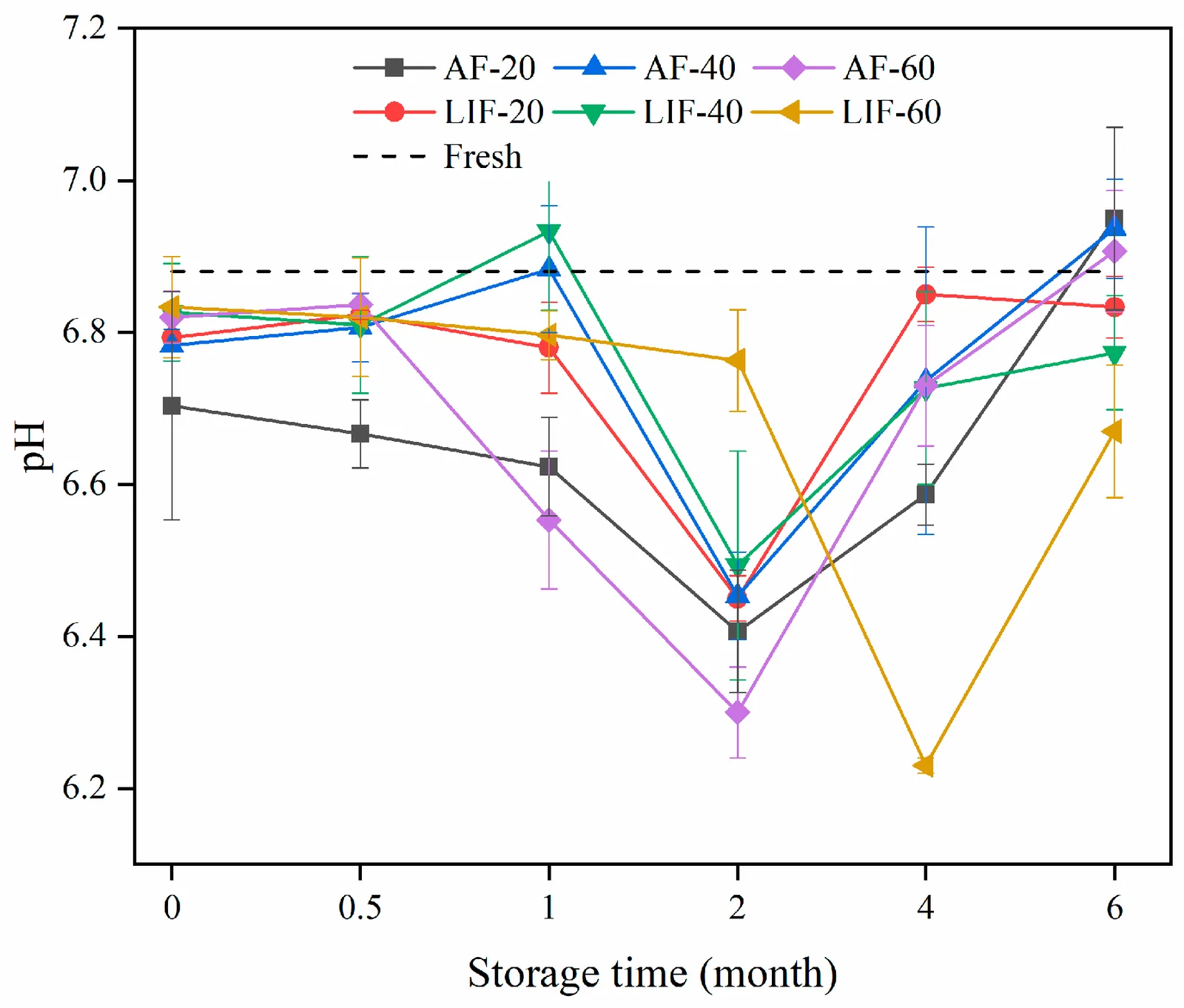
The pH of snakehead fillets frozen by air freezing (AF) and liquid immersion freezing (LIF) at different temperatures (−20 °C, −40 °C and −60 °C) during frozen storage. The results were expressed as the mean ± standard deviation (*n* = 3).

The pH of fish muscle is an important physicochemical indicator associated with changes in muscle metabolism and protein properties during processing and storage [17]. As shown in Figure 4, no significant differences in pH were observed among most freezing treatments immediately after freezing (0 months), except for the AF-20 group, which exhibited a relatively lower pH value. This difference may be associated with freezing-induced changes in muscle structure and the accumulation of acidic metabolites during postmortem metabolism. Compared with liquid immersion freezing, the relatively slower heat transfer during air freezing at −20 °C may contribute to greater structural changes in muscle tissue, potentially affecting the distribution of metabolites and the measured pH [6].

As shown in Figure 4, the pH of snakehead fillets varied during frozen storage, generally exhibiting an initial decrease followed by a subsequent increase, although the timing and magnitude of these changes differed among freezing treatments. The initial reduction in pH may be associated with changes in the balance of acidic metabolites and muscle buffering capacity during frozen storage [21]. In contrast, the subsequent increase in pH may be related to changes in protein-associated nitrogenous compounds and other biochemical processes occurring during prolonged storage. The different pH profiles among treatments may reflect variations in freezing-induced structural changes and subsequent biochemical stability. Notably, the LIF-60 group exhibited relatively stable pH values during the early storage period, suggesting that low-temperature liquid immersion freezing may help moderate physicochemical changes in fish muscle. These observations are consistent with previous findings on frozen largemouth bass [12].

### 3.4. Protein Secondary Structure

Fish contains a significant amount of protein ranging from 16 to 21% [20]. The structure of proteins significantly influences the structural quality of aquatic products such as their texture and water-holding capacity [11]. During freezing and subsequent frozen storage, protein denaturation is likely driven by ice crystal formation, dehydration, and the concentration of solutes within the cells [10]. Thus, monitoring protein structure is essential for maintaining the quality of the final product. Raman spectroscopy, with its high sensitivity to molecular vibrations, is commonly used to analyze the secondary structure of proteins in food. This technique is particularly valuable because Raman spectra are minimally influenced by the water content in the food [27]. Raman spectroscopy primarily analyzes protein structures by assessing amide conformations including the amide I band (1600–1700 cm^−1^), amide II band (1510–1560 cm^−1^) and amide III band (1230–1350 cm^−1^) [19]. Among these, the amide I band is most commonly used to assess protein secondary structures, as it is primarily associated with peptide C=O stretching vibrations, with contributions from other vibrational modes. The primary components of protein secondary structures are α-helix (1646–1664 cm^−1^), β-sheet (1615–1637 cm^−1^ and 1682–1700 cm^−1^), β-turn (1665–1681 cm^−1^), and random coil (1637–1645 cm^−1^) [30]. Among these, the content of α-helix and β-sheet serves as indicators of the regularity within protein structures. The stability of the α-helix is primarily influenced by hydrogen bonds between the amino (-NH) and carbonyl (-CO) groups in the peptide chain [24]. Changes in the Raman amide I region following freezing treatment indicated alterations in protein secondary structure, which may be associated with freezing-induced protein denaturation.

**Figure 5:**
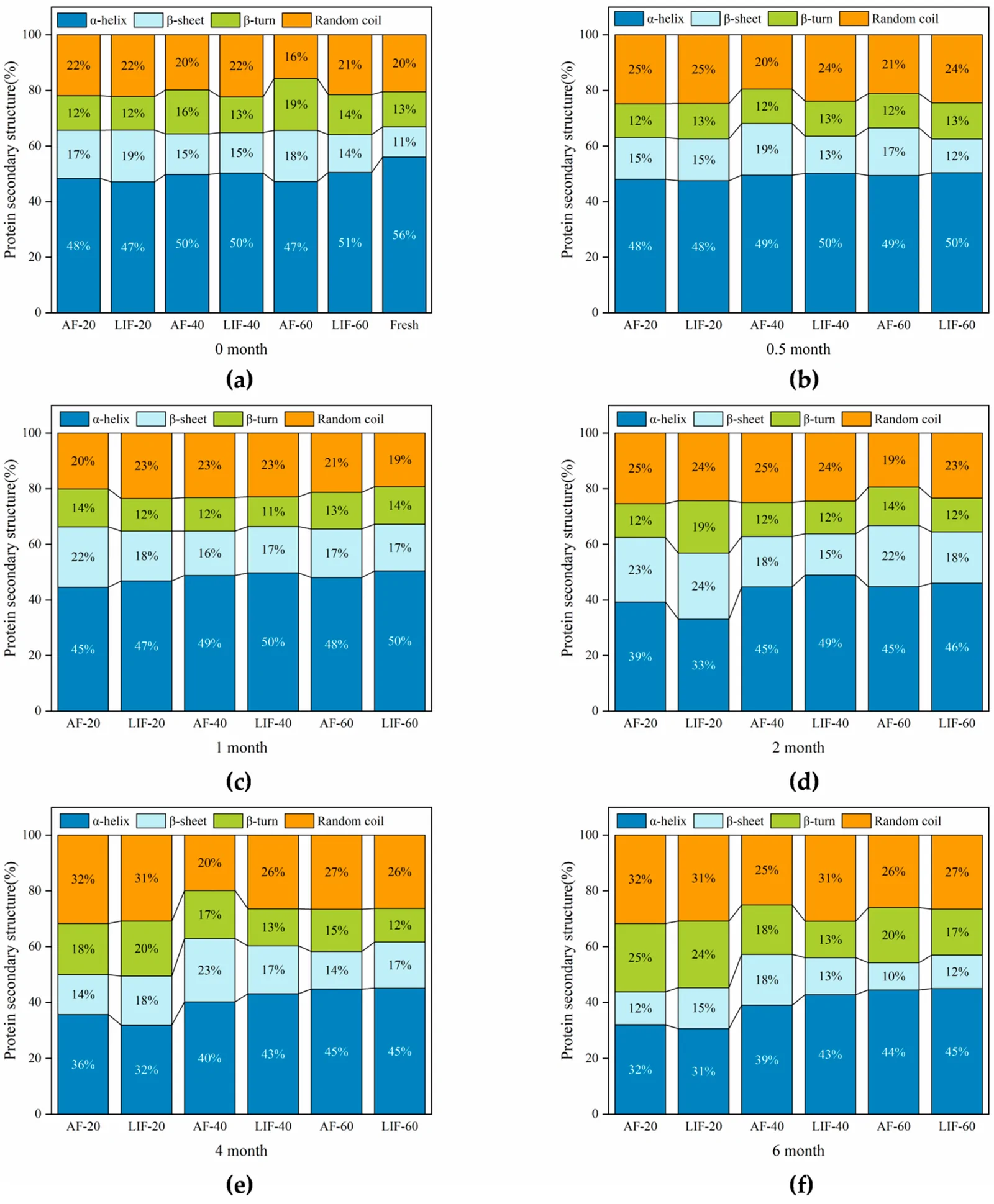
Protein secondary structure composition of snakehead fillets during frozen storage after different freezing treatments at (**a**) 0 months, (**b**) 0.5 months, (**c**) 1 month, (**d**) 2 months, (**e**) 4 months, and (**f**) 6 months. Each panel shows the proportions of alpha-helix, beta-sheet, beta-turn, and random coil structures in the AF-20, LIF-20, AF-40, LIF-40, AF-60, and LIF-60 groups; the fresh sample is additionally shown in panel (**a**). AF, air freezing; LIF, liquid immersion freezing. Values are means (*n* = 3).

As shown in Figure 5, the protein secondary structure of fresh snakehead fillets consisted of α-helix (56.05%), β-sheet (10.89%), β-turn (12.60%), and random coil (20.47%). Following different freezing treatments, noticeable changes in the content of these secondary structure components were observed in the fillets. Among all groups, samples of LIF-60, AF-40, and LIF-40 groups showed the lowest variations in secondary structure contents. Freezing-induced dehydration and freeze concentration may disrupt intramolecular interactions, expose hydrophobic groups, and destabilize the ordered secondary structure of proteins. These changes may alter hydrogen-bonding interactions that contribute to the stability of protein secondary structures [19]. As storage time progressed, the content of α-helix and β-sheet in snakehead fillets decreased, while the proportion of β-turn and random coil increased. From 0 to 1 month of storage, no noticeable changes were observed in the protein secondary structure of the fillets. However, between 1 and 2 months of storage, the α-helix content decreased markedly in several treatment groups, whereas the LIF-40 group showed only a small change, from approximately 50% to 49%. This may be attributed to the formation of small ice crystals in fillets subjected to −40 °C LIF treatment that resulted in minimal damage to cell structures. In contrast, the α-helix content in the LIF-20 group decreased markedly, from approximately 47% to 31%, over 6 months of frozen storage. This pronounced structural change may be associated with ice crystal growth and recrystallization during frozen storage, which can promote structural damage to proteins [24]. The decrease in α-helix and β-sheet contents indicated a transition from ordered to less ordered protein structures during frozen storage. This structural transition was consistent with the increased water loss and decreased hardness observed in the fillets. Therefore, ice crystal growth and recrystallization during storage may contribute to myofibrillar protein destabilization, reduced water-holding capacity, and texture deterioration.

### 3.5. Microstructure

Ice crystal size plays a crucial role in determining the final quality of frozen aquatic products [25]. The freezing process occurs in three stages. The first stage, known as the pre-cooling stage, typically spans from 4 °C to 0 °C, during which the sample loses sensible heat. The second stage, referred to as the phase transition stage (usually from 0 °C to −5 °C), is when the maximum formation of ice crystals occurs. During this phase, latent heat is removed, and a substantial amount of liquid water solidifies into ice [23]. The longer this phase transition phase is, the slower the rate of freezing and the higher the possibility of forming large extracellular ice crystals in the sample, which can damage the quality of cells [31]. The third stage, known as the deep freezing or tempering stage, typically occurs between −5 °C and −20 °C. During this phase, the remaining water in the sample is rapidly frozen, ultimately lowering the temperature to its final point [5].

**Figure 6:**
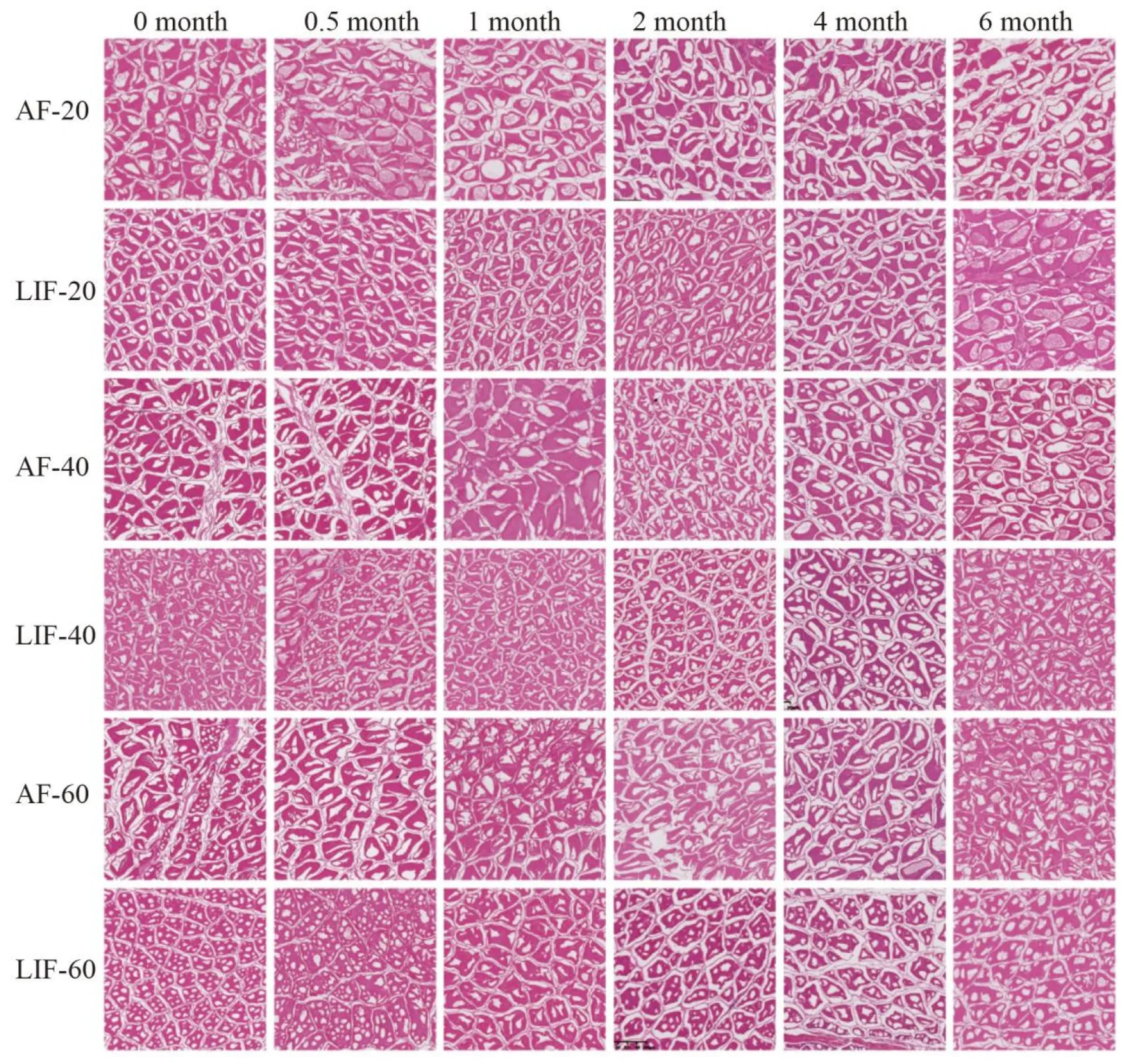
Microstructure of snakehead fillets subjected to different freezing treatments during frozen storage. Rows represent AF-20, LIF-20, AF-40, LIF-40, AF-60, and LIF-60, and columns represent storage times of 0, 0.5, 1, 2, 4, and 6 months. AF, air freezing; LIF, liquid immersion freezing; 20, 40, and 60 indicate freezing temperatures of −20, −40, and −60 °C, respectively.

As shown in Table 2 and Figure 6, after freezing (0 months), the largest ice crystal area was observed in the AF-20 group, while the smallest was found in the LIF-60 group. Under the same freezing conditions, the LIF samples exhibited significantly smaller ice crystal areas (*p* < 0.05) compared to the AF samples. Overall, lower freezing temperatures tended to reduce ice crystal size. Studies have shown that the heat-transfer coefficient for liquid immersion freezing is 100–140 W/(m^2^⋅K), which is approximately ten times greater than that of air freezing, which ranges from 15 to 17 W/(m^2^⋅K) [9,32]. Furthermore, a lower coolant temperature increases the temperature difference driving heat transfer, which may contribute to the formation of smaller ice crystals, consistent with the morphological observations in this study. Accordingly, the LIF-60 group exhibited the smallest ice crystal area immediately after freezing.

The results for ice crystal diameter were similar to those for the ice crystal area. As shown in Table 2, after freezing, the ice crystal diameter of AF-20 was significantly (*p* < 0.05) larger compared to other treatment groups. By contrast, the ice crystal diameter of LIF-60 group was significantly (*p* < 0.05) smaller compared to samples from other freezing groups. Compared with LIF, air freezing generally provides less efficient heat transfer, which may contribute to the formation of larger ice crystals, particularly in the AF-20 group. These large extracellular ice crystals can be expected to disrupt cell integrity which, in turn, enhances moisture loss in the samples. Additionally, large extracellular ice crystals also lead to protein denaturation and decreased hardness of sample under investigation [15,25].

Table 2 shows that ice crystal area and equivalent diameter generally increased during frozen storage, although some fluctuations were observed among treatments and storage periods. After 6 months, the AF-20 group exhibited the largest mean ice crystal area (385.94 μm^2^) and equivalent diameter (35.28 μm), whereas the LIF-60 group showed the smallest mean values (105.54 μm^2^ and 15.34 μm, respectively). Although the ice crystal area of LIF-60 was numerically smaller than that of LIF-40 (117.39 μm^2^), no significant difference was observed between these treatments (*p* > 0.05). In contrast, their equivalent diameters differed significantly (*p* < 0.05). These findings indicate that liquid immersion freezing at lower temperatures effectively limited ice crystal enlargement during frozen storage. The relatively small ice crystals observed in LIF-40 and LIF-60 may contribute to the preservation of muscle structure and water-holding capacity. Compared with ice crystal area and equivalent diameter, elongation and roundness showed less consistent trends among treatments and storage times. Therefore, cross-sectional area and equivalent diameter were considered more informative indicators for evaluating freezing-induced structural changes in snakehead fillets.

**Figure 7:**
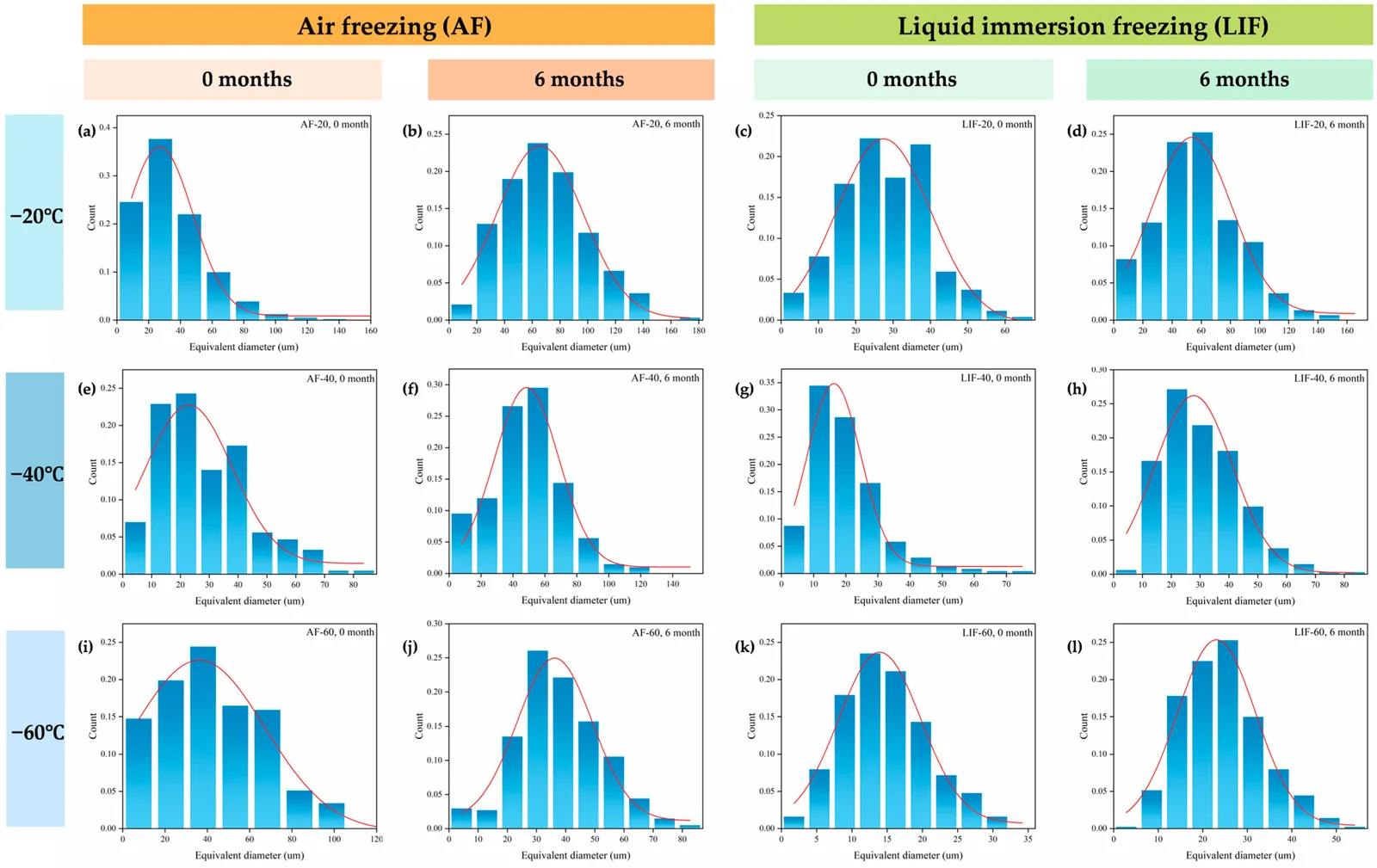
Distributions of equivalent ice crystal diameter in snakehead fillets subjected to different freezing treatments at 0 and 6 months of frozen storage: (**a**) AF-20 at 0 months; (**b**) AF-20 at 6 months; (**c**) LIF-20 at 0 months; (**d**) LIF-20 at 6 months; (**e**) AF-40 at 0 months; (**f**) AF-40 at 6 months; (**g**) LIF-40 at 0 months; (**h**) LIF-40 at 6 months; (**i**) AF-60 at 0 months; (**j**) AF-60 at 6 months; (**k**) LIF-60 at 0 months; and (**l**) LIF-60 at 6 months. AF, air freezing; LIF, liquid immersion freezing; 20, 40, and 60 indicate freezing temperatures of −20, −40, and −60 °C, respectively.

As shown in Table 2 and Figure 7, the mean equivalent ice crystal diameter was generally greater after 6 months of frozen storage than immediately after freezing. Immediately after freezing, the equivalent ice crystal diameters in the AF-20 group were most frequently distributed within the range of 20–40 μm, whereas after 6 months of storage, the distribution shifted toward larger diameters, including the range of 60–80 μm. In the LIF-60 group, equivalent ice crystal diameters were most frequently distributed within the range of 10–15 μm immediately after freezing, and the predominant range shifted to 20–30 μm after 6 months of storage. Similar trends were observed in the other treatment groups. Ice recrystallization during frozen storage may contribute to the observed enlargement of ice crystal cavities [26]. Differences in thermal history, including the transfer of samples from their initial freezing temperatures to −30 °C storage, may also influence subsequent structural changes [21]. Larger ice crystal cavities may be associated with greater muscle tissue disruption and changes in water-holding capacity. These findings are in agreement with previous results observed in case of obscure pufferfish fillets subjected to immersion freezing [15].

### 3.6. PCA

Principal component analysis (PCA) was used to characterize multivariate variation in the quality of snakehead fillets across different freezing treatments and storage periods. Data on thawing loss, cooking loss, total weight loss, α-helix content, ice crystal area, and ice crystal diameter were included in the PCA. The first two principal components (PC1 and PC2) explained 89.2% of the total variance, capturing the major patterns of variation among the six quality indicators.

**Figure 8:**
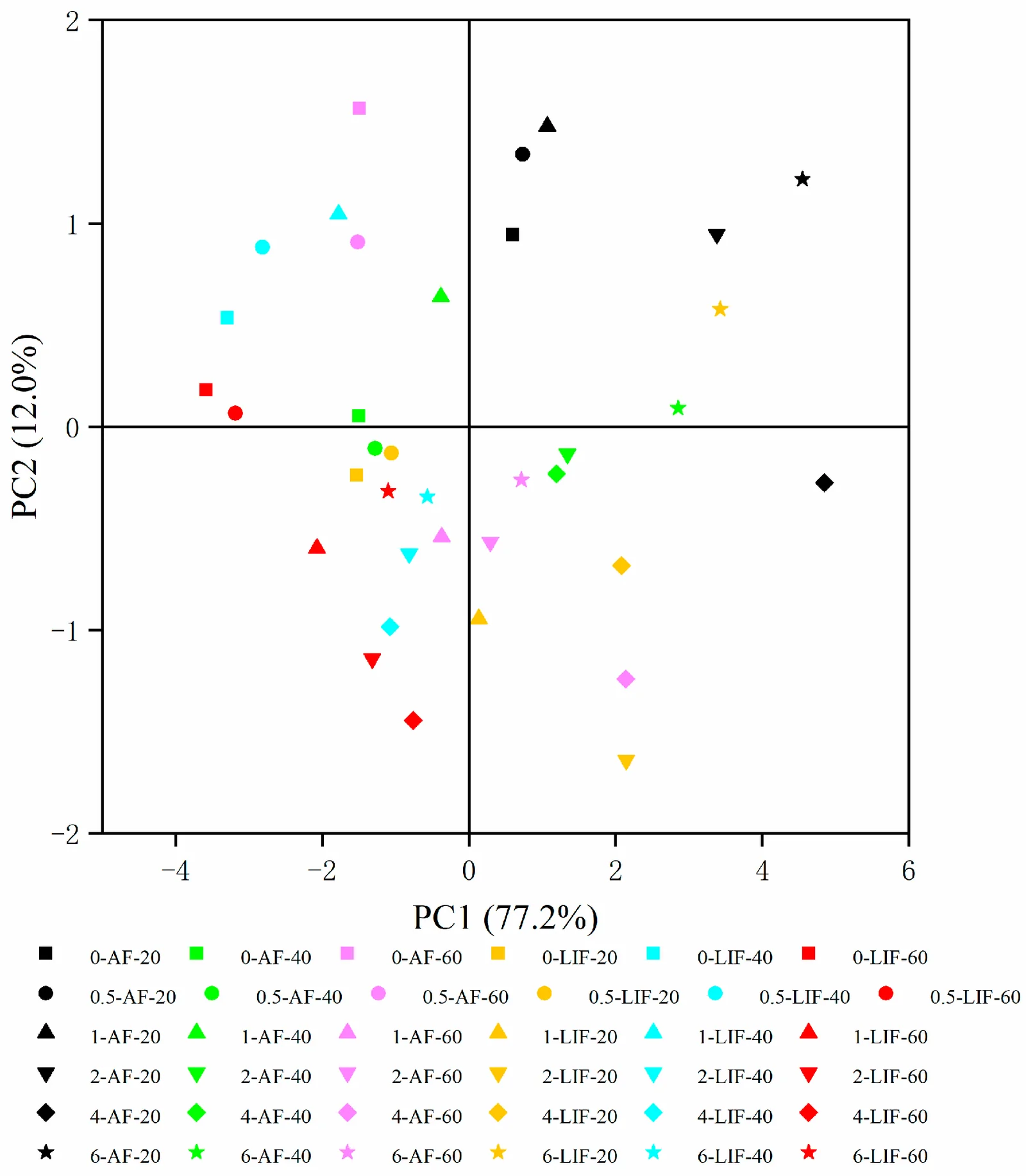
Principal component analysis (PCA) of the properties of snakehead fillets frozen by air freezing (AF) and liquid immersion freezing (LIF) at −20 °C, −40 °C and −60 °C during frozen storage.

The scores for two PCs of frozen snakehead fillets with different freezing methods and different storage times are shown in Figure 8. Based on the freezing method, samples of the AF-20 group were distributed on the positive half axis of PC1, and samples of the LIF-60 group were distributed on the negative half axis of PC1, clearly separating the two samples. The samples from different storage times were scattered in the score plot according to the variation in their quality. Samples with storage times of 0 and 0.5 months were mainly distributed in the negative half axis of PC1 (except AF-20). The samples LIF-60 and LIF-40 stored for 6 months were also distributed in the negative half axis of PC1 and the remaining samples were distributed in the positive half axis of PC1. The PCA score plot suggested that quality differences among treatments began to become distinguishable during the early stage of frozen storage. In particular, the LIF-40 and LIF-60 samples showed score distributions that were distinct from those of AF-20, reflecting differences in the measured quality attributes during frozen storage. These results indicate that appropriate LIF conditions can help maintain the frozen storage quality of snakehead fillets.

## 4. Conclusions

In this study, air freezing and liquid immersion freezing at −20 °C, −40 °C, and −60 °C were applied to snakehead fillets, followed by frozen storage at −30 °C for 6 months. Freezing and frozen storage caused increased water loss, reduced hardness, changes in pH, alterations in protein secondary structure, and enlargement of ice crystals. AF-20 resulted in the greatest quality deterioration, as reflected by higher water loss, lower hardness, and larger ice crystal area and diameter. In contrast, LIF treatments, especially LIF-40 and LIF-60, better maintained the quality of snakehead fillets during frozen storage. LIF-60 generally produced smaller ice crystals, whereas LIF-40 also showed favorable water retention after prolonged frozen storage. These results suggest that liquid immersion freezing at appropriately low temperatures can improve the frozen storage stability of snakehead fillets by reducing ice crystal damage and slowing protein structural deterioration. The findings provide useful information for selecting freezing conditions for frozen snakehead fillet processing.

## Figures and Tables

**Table 1:** Thawing, cooking and total weight loss of snakehead fillets frozen by air freezing and liquid immersion freezing during storage.

Thawing loss (%)						
	0 month	0.5 month	1 month	2 month	4 month	6 month
AF-20	1.05 ± 0.3 ^a^	1.06 ± 0.26 ^a^	1.07 ± 0.19 ^abc^	2.24 ± 0.32 ^a^	2.43 ± 0.61 ^a^	1.78 ± 0.03 ^a^
LIF-20	0.38 ± 0.1 ^c^	0.53 ± 0.09 ^bc^	1.1 ± 0.01 ^ab^	1.21 ± 0.21 ^b^	1.17 ± 0.26 ^bc^	0.75 ± 0.12 ^c^
AF-40	0.81 ± 0.02 ^b^	0.97 ± 0.02 ^a^	1.13 ± 0.37 ^a^	1.15 ± 0.16 ^b^	1.16 ± 0.16 ^bc^	1.27 ± 0.06 ^b^
LIF-40	0.26 ± 0.06 ^c^	0.35 ± 0.04 ^c^	0.62 ± 0.14 ^bc^	0.65 ± 0.05 ^c^	0.65 ± 0.07 ^c^	0.52 ± 0.14 ^c^
AF-60	0.54 ± 0.19 ^bc^	0.61 ± 0.14 ^b^	1.1 ± 0.39 ^ab^	1.15 ± 0.07 ^b^	1.44 ± 0.08 ^b^	1.05 ± 0.07 ^b^
LIF-60	0.29 ± 0.04 ^c^	0.36 ± 0.03 ^c^	0.57 ± 0.16 ^c^	0.66 ± 0.17 ^c^	0.79 ± 0.06 ^c^	0.67 ± 0.16 ^c^
Cooking loss (%)						
	0 month	0.5 month	1 month	2 month	4 month	6 month
AF-20	16.79 ± 0.96 ^a^	16.77 ± 1.04 ^a^	16.54 ± 0.98 ^a^	17.94 ± 2.76 ^a^	20.73 ± 0.9 ^a^	19.18 ± 1.65 ^a^
LIF-20	15.92 ± 0.44 ^a^	16.41 ± 0.75 ^ab^	17.87 ± 0.23 ^a^	19.84 ± 2.79 ^a^	18.32 ± 1.21 ^abc^	19.69 ± 0.73 ^a^
AF-40	15.53 ± 1.4 ^ab^	15.94 ± 2.7 ^ab^	16.5 ± 1.22 ^a^	18.44 ± 1.87 ^a^	17.53 ± 1.45 ^c^	19.57 ± 0.08 ^a^
LIF-40	13.01 ± 0.95 ^c^	13.46 ± 0.35 ^b^	14.3 ± 0.79 ^b^	17.3 ± 0.08 ^a^	16.68 ± 1.43 ^c^	16.84 ± 1.27 ^b^
AF-60	13.77 ± 0.72 ^bc^	14.35 ± 0.74 ^ab^	17.03 ± 2.15 ^a^	17.87 ± 1.54 ^a^	20.41 ± 1.47 ^ab^	17.92 ± 0.57 ^ab^
LIF-60	12.93 ± 1.32 ^c^	13.95 ± 1.2 ^b^	15.92 ± 0.53 ^ab^	16.84 ± 1.92 ^a^	18 ± 0.76 ^bc^	16.43 ± 0.53 ^b^
Fresh	12.43 ± 0.5 ^c^					
Total weight loss (%)						
	0 month	0.5 month	1 month	2 month	4 month	6 month
AF-20	17.89 ± 1.25 ^a^	17.33 ± 3.12 ^a^	17.43 ± 1.11 ^ab^	19.77 ± 2.94 ^a^	22.39 ± 1.37 ^a^	20.61 ± 2.48 ^a^
LIF-20	16.58 ± 0.16 ^ab^	16.94 ± 0.94 ^a^	19.11 ± 1.2 ^a^	20.81 ± 2.76 ^a^	19.99 ± 2.89 ^abc^	20.3 ± 0.63 ^a^
AF-40	16.03 ± 0.7 ^bc^	16.15 ± 3.16 ^a^	15.93 ± 2.77 ^ab^	19.6 ± 1.77 ^a^	19.27 ± 0.99 ^bc^	20.58 ± 0.03 ^a^
LIF-40	13.92 ± 1.15 ^d^	13.82 ± 0.48 ^a^	14.83 ± 0.84 ^b^	17.82 ± 0.05 ^a^	17.11 ± 1.56 ^ac^	17.26 ± 1.39 ^b^
AF-60	14.68 ± 0.34 ^cd^	15.81 ± 0.73 ^a^	17.94 ± 2.42 ^ab^	18.09 ± 1.99 ^a^	21.55 ± 1.39 ^b^	18.78 ± 0.51 ^ab^
LIF-60	13.95 ± 0.52 ^d^	14.19 ± 1.29 ^a^	16.39 ± 0.66 ^ab^	17.49 ± 1.95 ^a^	18.4 ± 0.77 ^c^	16.99 ± 0.65 ^b^
Fresh	12.43 ± 0.5 ^d^					

The results were expressed as the mean ± standard deviation *(n* = 3), and the letters (a–d) in the same column indicated significant differences (*p* < 0.05) between the data of different samples. Samples were frozen at −20 °C, −40 °C and −60 °C. Note: AF, air freezing; LIF, liquid immersion freezing.

**Table 2:** Microscopic analysis of ice crystals in snakehead fillets frozen by air freezing and liquid immersion freezing at −20 °C, −40 °C and −60 °C.

Storage Time	Treatment	Cross-Sectional Area (μm^2^)	Equivalent Diameter (μm)	Elongation	Roundness
0 month	AF-20	263.19 ± 170.69 ^a^	23.29 ± 8.62 ^a^	6.4 ± 3.17 ^c^	0.31 ± 0.21 ^bc^
	LIF-20	86.06 ± 48.51 ^c^	15.33 ± 5.52 ^c^	7.67 ± 3.89 ^b^	0.32 ± 0.18 ^bc^
	AF-40	96.3 ± 70.14 ^c^	15.22 ± 6.97 ^c^	7.7 ± 5.29 ^b^	0.31 ± 0.17 ^c^
	LIF-40	58.99 ± 52.70 ^d^	11.35 ± 5.48 ^d^	6.48 ± 4.27 ^c^	0.36 ± 0.19 ^ab^
	AF-60	176.77 ± 131.56 ^b^	20.56 ± 9.64 ^b^	9.18 ± 6.05 ^a^	0.29 ± 0.25 ^c^
	LIF-60	32.89 ± 22.50 ^e^	8.64 ± 3.40 ^e^	5.99 ± 2.64 ^c^	0.39 ± 0.14 ^a^
0.5 month	AF-20	282.46 ± 181.15 ^a^	25.47 ± 9.56 ^a^	8.15 ± 4.99 ^abc^	0.28 ± 0.25 ^c^
	LIF-20	100.1 ± 55.09 ^c^	17.77 ± 5.7 ^b^	9.22 ± 5.31 ^a^	0.26 ± 0.16 ^c^
	AF-40	92.51 ± 60.76 ^c^	15.05 ± 6.03 ^c^	7.23 ± 4.53 ^c^	0.34 ± 0.15 ^b^
	LIF-40	84.46 ± 74.24 ^c^	14.87 ± 7.84 ^c^	8.09 ± 5.32 ^bc^	0.29 ± 0.20 ^c^
	AF-60	140.5 ± 104.3 ^b^	18.75 ± 8.51 ^b^	8.71 ± 5.7 ^ab^	0.28 ± 0.16 ^c^
	LIF-60	48.46 ± 42.63 ^d^	9.58 ± 4.33 ^d^	4.94 ± 2.17 ^d^	0.45 ± 0.15 ^a^
1 month	AF-20	317.39 ± 213.26 ^a^	25.32 ± 10.59 ^a^	6.48 ± 4.92 ^ab^	0.32 ± 0.20 ^b^
	LIF-20	114.35 ± 78.09 ^c^	16.44 ± 6.80 ^cd^	7.68 ± 5.63 ^a^	0.31 ± 0.15 ^b^
	AF-40	175.31 ± 105.75 ^b^	19.89 ± 6.39 ^b^	6.76 ± 4.04 ^ab^	0.32 ± 0.16 ^b^
	LIF-40	137.96 ± 91.08 ^c^	18.14 ± 7.61 ^bc^	6.09 ± 4.5 ^b^	0.43 ± 0.27 ^a^
	AF-60	123.83 ± 106.3 ^c^	15.37 ± 7.63 ^d^	5.65 ± 3.24 ^b^	0.37 ± 0.27 ^b^
	LIF-60	59.57 ± 60.57 ^d^	11.36 ± 6.24 ^e^	6.91 ± 3.74 ^ab^	0.34 ± 0.18 ^b^
2 month	AF-20	331.35 ± 188.08 ^a^	28.58 ± 9.96 ^a^	6.51 ± 3.72 ^ab^	0.26 ± 0.13 ^c^
	LIF-20	157.1 ± 102.89 ^c^	18.14 ± 6.79 ^c^	6.99 ± 6.07 ^ab^	0.31 ± 0.14 ^b^
	AF-40	180.2 ± 124.18 ^b^	24.94 ± 10.82 ^b^	6.91 ± 5.70 ^ab^	0.2 ± 0.14 ^d^
	LIF-40	94.72 ± 62.77 ^d^	16.44 ± 6.35 ^d^	6.47 ± 3.80 ^b^	0.3 ± 0.11 ^b^
	AF-60	137 ± 115.58 ^c^	17.69 ± 8.19 ^cd^	7.45 ± 4.27 ^a^	0.31 ± 0.15 ^b^
	LIF-60	59.46 ± 38.00 ^e^	11.07 ± 3.91 ^e^	5.08 ± 2.23 ^c^	0.44 ± 0.14 ^a^
4 month	AF-20	359.21 ± 199.96 ^a^	27.03 ± 9.08 ^a^	7.8 ± 5.98 ^ab^	0.28 ± 0.14 ^d^
	LIF-20	183.12 ± 87.04 ^b^	21.92 ± 6.47 ^b^	8.47 ± 5.35 ^a^	0.27 ± 0.13 ^d^
	AF-40	189.76 ± 130.88 ^b^	21.06 ± 7.89 ^b^	7.48 ± 4.48 ^b^	0.33 ± 0.17 ^c^
	LIF-40	98.96 ± 78.11 ^c^	10.14 ± 3.78 ^d^	6.42 ± 3.83 ^c^	0.73 ± 0.30 ^a^
	AF-60	195.43 ± 144.33 ^b^	20.92 ± 9.02 ^b^	7.49 ± 4.78 ^b^	0.32 ± 0.18 ^c^
	LIF-60	70.02 ± 45.07 ^d^	11.94 ± 4.19 ^c^	4.81 ± 2.22 ^d^	0.47 ± 0.15 ^b^
6 month	AF-20	385.94 ± 257.10 ^a^	35.28 ± 11.83 ^a^	8.53 ± 4.75 ^a^	0.19 ± 0.08 ^e^
	LIF-20	383.03 ± 262.56 ^a^	29.11 ± 11.64 ^b^	8.19 ± 3.97 ^ab^	0.28 ± 0.14 ^d^
	AF-40	312.65 ± 196.72 ^b^	26.14 ± 10.34 ^c^	7.21 ± 3.49 ^c^	0.33 ± 0.21 ^b^
	LIF-40	117.39 ± 70.45 ^d^	17.23 ± 6.18 ^e^	7.11 ± 3.54 ^c^	0.32 ± 0.13 ^bc^
	AF-60	171.55 ± 89.15 ^c^	20.83 ± 6.25 ^d^	7.6 ± 3.85 b^c^	0.29 ± 0.23 ^cd^
	LIF-60	105.54 ± 65.90 ^d^	15.34 ± 5.17 ^f^	5.28 ± 1.99 ^d^	0.44 ± 0.13 ^a^

Values are presented as mean ± standard deviation. Different lowercase letters within the same storage period and parameter indicate significant differences among freezing treatments (*p* < 0.05).

## Data Availability

The original contributions presented in this study are included in the article. Further inquiries can be directed to the corresponding author.
